# Biotransformation of Flaxseed Oil Cake into Bioactive Camembert-Analogue Using Lactic Acid Bacteria, *Penicillium camemberti* and *Geotrichum candidum*

**DOI:** 10.3390/microorganisms8091266

**Published:** 2020-08-20

**Authors:** Łukasz Łopusiewicz, Emilia Drozłowska, Alicja Tarnowiecka-Kuca, Artur Bartkowiak, Kinga Mazurkiewicz-Zapałowicz, Piotr Salachna

**Affiliations:** 1 Center of Bioimmobilisation and Innovative Packaging Materials, Faculty of Food Sciences and Fisheries, West Pomeranian University of Technology in Szczecin, Janickiego, 35, 71-270 Szczecin, Poland; emilia_drozlowska@zut.edu.pl (E.D.); alicja.tarnowiecka-kuca@zut.edu.pl (A.T.-K.); artur-bartkowiak@zut.edu.pl (A.B.); 2 Department of Hydrobiology, Ichthyology and Biotechnology of Reproduction, West Pomeranian University of Technology in Szczecin, Kazimierza Królewicza, 4, 71-899 Szczecin, Poland; Kinga.Mazurkiewicz-Zapalowicz@zut.edu.pl; 3 Department of Horticulture, West Pomeranian University of Technology in Szczecin, Papieża Pawła, VI 3, 71-459 Szczecin, Poland; piotr.salachna@zut.edu.pl

**Keywords:** flaxseed, oil cake, vegan product, cheese analogue, functional foods, antioxidant activity, *Penicillium camemberti*, *Geotrichum candidum*, lactic acid bacteria

## Abstract

This study aimed at investigating the antioxidant activity, oxidative stability, physicochemical and microbial changes of innovative vegan Camembert-analogue based on flaxseed oil cake (FOC) which was produced using lactic acid bacteria (LAB), mold *Penicillium camemberti* (PC) and yeast *Geotrichum candidum* (GC). Two variants were prepared, namely with LAB + PC and LAB + PC + GC. After fermentation for 24 h at room temperature, the samples were stored for 14 days at 12 °C and maturated for 14 days at 6 °C. Changes in microbial population, polyphenolics, flavonoids, radical scavenging capacity were evaluated. Additionally, textural changes, pH, acidity, levels of proteins, free amino acids, reducing sugars, oil content and its oxidative stability were determined. Results showed that LAB as well as fungi were capable of growing well in the FOC without any supplementation and the products were characterized by a high antioxidant potential (high polyphenolics and flavonoids contents as well as 2,2-diphenyl-1-picrylhydrazyl (DPPH), 2,2′-azino-bis(3-ethylbenzothiazoline-6-sulfonic acid) (ABTS), superoxide (O_2_^−^) and hydroxyl (^·^OH) radicals scavenging activity). This study has demonstrated that bioactivity as well as the physicochemical properties depend on the starter culture used. Due to functional and biochemical characteristics conferred to the obtained Camembert-analogues, the use of *P. camemberti* and *G. candidum* showed a potential for industrial application. There is a potential for these products to be used where non-dairy alternatives are desired.

## 1. Introduction

In the recent years, vegetarian and vegan diets have become more widespread because of a higher attention of consumers to nutritional, ethical, and cultural aspects [1,2,3]. Moreover, consumers demand for cows’ milk alternatives increased as a result of the increase in the diagnosis of lactose intolerance, allergies and cholesterol issues [1,4,5]. Not wishing to consume food of animal origin, vegan consumers are looking for substitutes that could enrich their diet and contribute to a good health [2]. Therefore, a need has arisen to offer consumers an alternative to fermented dairy products by exploring new non-dairy matrices [3]. The search for new functional food ingredients from natural sources and from by-products represents one of the most important challenges in food science and technology. Food industry by-products represent a valuable source of minerals, proteins, fatty acids, fiber, and bioactive substances, and they can constitute as an important raw material for the development of novel functional foods [6,7,8]. Simultaneously, the intake of plant-based products, which are rich sources of bioactive compounds, increases notably [9,10]. In general, plant products are known for their contents of phytochemicals (e.g., polyphenols such as phenolic acids and flavonoids), bioactive peptides or other bioactive constituents which exhibit therapeutic properties such as anti-microbial, anti-inflammatory, anti-thrombotic, anti-allergenic, anti-atherogenic, antioxidant, anti-cardiovascular disease and vasodilatory effects [11,12]. This is of particular importance, because unhealthy lifestyle, intense physical exercising, stress, and environmental pollution are factors that influence the excessive synthesis of reactive oxygen species, which may disturb the homeostasis of human body and lead to the formation of oxidative stress [13].

Fermentation is one of the oldest food technology applications. Fermented products are the result of the metabolic activity of a complex microbiota, consisting of the naturally occurring indigenous microorganisms, and/or selected microorganisms such as bacteria, molds and yeasts which inoculated as starter cultures [1,11,14]. Among fermented foods, fermented products from either dairy or non-dairy origin play an important role in the human diet around the world [5]. The popularity of fermented foods and beverages is due to their enhanced shelf-life, safety, functionality, sensory, and nutritional properties. The latter includes the presence of bioactive molecules, vitamins, and other constituents with increased availability due to the process of fermentation. Fermented foods account for about 25% of diets in Europe and 60% in developing countries [6]. Especially in the western diet, fermented milk products (in which yogurt, kefir and cheese remain at the forefront of dairy products) represent 60% of fermented foods [9]. Cheese (which is rich in proteins and fat) is traditionally a dairy product, meaning it is derived from milk (cow, goat, or sheep), processed in different ways (mainly by metabolic action of the microorganisms already present or added to milk), and allowed to mature under certain conditions for certain period of time [15,16]. During ripening, biochemical reactions (proteolysis, lipolysis and glycolysis) associated with the particular flavor, texture and bioactivity of each cheese variety, are carried out by enzymes either produced by live microorganisms or released onto the cheese matrix after microbial cellular lysis [15].

Cheese-like products (or cheese analogues) are usually defined as products made by blending individual components, including non-dairy fats or proteins, to produce a cheese-like product to meet specific requirements. They are increasingly used due to their cost-effectiveness, which is attributable to the simplicity of their production and the replacement of selected milk ingredients by cheaper vegetable products [17]. Vegan cheeses are entirely plant-based and involve consolidating the protein mass from various plant sources with lactic bacteria that may also be added in for acidity. Oils, emulsifiers, and thickeners are also often used to produce firmer types of vegan cheeses. “Cheese-like” products made from soy (such as tofu) or nuts are one of the most popular vegan products. The “nut vegan cheeses” are made from peanuts, cashews, macadamias, almonds, or other nuts by soaking and grinding with water in some cases followed by fermentation [2,14,18,19,20]. However some other plant sources such as rice flour [21], apricot pulp [22] and sweet corn extract [23] have been also considered. One of the most known ripened cheeses is Camembert—a soft, high humidity cheese which is covered with a velvety white layer originated mainly by the growth of the mold *Penicillium camemberti* (sometimes with participation of yeast *Geotrichum candidum*), and presents, on average, 19.8% protein, 24.3% fat and 51.8% moisture [24]. Its ripening shows great complexity especially due to an intense proteolysis which is affected by the action of the residual coagulant, natural milk enzymes (especially when raw milk is used), enzymes from the acidifying starter culture, non-starter lactic acid bacteria, and also by the enzymes produced by the fungi, which have intense proteolytic activity and give Camembert cheese its specific flavor, characteristic aroma and bioactivity [24,25]. In fact, it is commonly accepted that Camembert cheese proteolytic activity is of microbial origin, especially from the mold *P. camemberti*, whereas the deamination activities of come bacteria and yeast such as *G. candidum* lead to formation of ammonia, aldehydes and organic acids that have a role in cheese flavor [26,27,28,29,30]. Although the literature reports on the use of certain plant matrices (e.g., from peanut [2]) to develop ripened cheese-like products, the reports about involving of mold *P. camemberti* and yeast *G. candidum* for production of ripened cheese-like products based on plant matrices and the evaluation of their bioactivity and physicochemical properties during ripening are still scarce.

Oil cakes are by-products obtained after pressing of oil from seeds, which have a high nutritional value as their protein content ranges from 15% to 50%, and low caloric value due to low oil level [31,32]. Due to their high protein content, they are used as animal feed supplement, in particular for ruminants and fish. However, their application in development of innovative vegan food products is rather limited. Flaxseed oil cake (FOC) is a protein-rich and cheap by-product of flaxseed (*Linum usitatissimum* L.) oil pressing [12]. It is a source of many bioactive substances such as α-linolenic acid (ALA), proteins, dietary fiber, phenolic compounds, and lignans. Despite the extraction of the beneficial flaxseed oil majority, FOC still has some oil content and a great nutritional potential. The removal of oil content increase the protein and fiber content in FOC. More importantly, FOC contains a concentrated amount of lignans, which are not extracted along with the flaxseed oil [12]. FOC may also contain antioxidant compounds, which are commonly found in whole flaxseed [12,33]. Many studies reported positive influence of flaxseed consumption regarding, e.g., colon cancer prevention, anti-inflammatory activity and reduction of the risk of cardiovascular diseases [12,34,35,36]. It is considered a “superfood” and GRAS (Generally Recognized as Safe) and is a plant food that meets the needs of the 21st century consumers in terms of being rich in nutrients as well as in bioactive and functional ingredients. FOC (after thermal processing related to reduction of cyanogenic compounds to a level that is safe for consumers) was found to be suitable raw material for development of new vegan food products including spray-dried powders with emulsifying activity [37], fermented yogurt-like plant milk [38] and kefir-like beverage [32]. However, due to high ALA content in flaxseed oil, it is a great challenge for the development of flaxseed based products.

To the best of our knowledge there have been no reports about utilization of flaxseed oil cake obtained via cold press technique to produce vegan analogue of Camembert cheese. Moreover, there are no reports on the physicochemical and microbial changes that occur during aging of such matrices. Thus, the aim of the presented study is to produce fermented products involving lactic acid bacteria, *P. camemberti*, *G. candidum* and evaluation of their bioactivity, oxidative stability, microbiological, and physicochemical properties during ripening for four weeks.

## 2. Materials and Methods

### 2.1. Materials and Reagents

Flaxseed oil cake (FOC) obtained via cold press technique was kindly donated by ACS Sp. z o.o. (Bydgoszcz, Poland). According to manufacturer’s information the proximate composition of FOC was: solids content 80.50%, ash content 4.50%, protein content 41.97%, fat content 6.11%, carbohydrates 27.99%, fiber 6.29%. Commercial starter cultures: PC^®^ (containing *Penicillium camemberti*), GEO^®^ (containing *Geotrichum candidum*) and MST Cheese-Tek^®^ (containing *Lactococcus lactis* subsp. *lactis*, *Lactococcus lactis* subsp. *cremoris* and *Streptococcus salivarius* subsp. *thermophilus*) were obtained from Biochem s.r.l. (Biochem s.r.l, Rome, Italy). Sodium hydroxide, sulfuric acid, boric acid, hydrogen peroxide, disodium phosphate, monosodium phosphate, phenolphthalein, 2,2-diphenyl-1-picrylhydrazyl (DPPH), 2,2′-azino-bis(3-ethylbenzothiazoline-6-sulfonic acid) (ABTS), methanol, Folin–Ciocalteu’s reagent, sodium carbonate, gallic acid, sodium nitrite, aluminum chloride, quercetin, 3,5-dinitrosalicylic acid, sodium tartrate tetrahydrate, acetic acid, sodium acetate, potassium ferricynide, trichloroacetic acid, ferric chloride, ninhydrin, glacial acetic acid, cadmium chloride, tris(hydroxymethyl)aminomethane, pyrogallol, ortophenantroline, glycine, p-anisidine, sodium thiosulfate, iron (II) sulphate, mercury (II) chloride and tin (II) chloride were purchased from Sigma Aldrich (Sigma Aldrich, Darmstadt, Germany). Glucose, hydrochloric acid, chloroform, Wijs reagent, ammonium thiocyanate and ethanol were supplied from Chempur (Chempur, Piekary Śląskie, Poland). Kjehdahl tablets (according to Missouri) were purchased from Carl Roth GmbH (Carl Roth GmbH, Karlsruhe, Germany). All reagents were of analytical grade. MRS agar, and Rose Bengal agar were obtained from Merck (Merck, Darmstadt, Germany).

### 2.2. Products Preparation, Fermentation and Ripening

The preparation of the samples consisted of few steps. Firstly, 700 g of FOC was mixed with 2000 mL of sterile distilled water and soaked at room temperature for 4 h. Then, the mixture was pasteurized (60 °C, 30 min), cooled down to room temperature and divided into two batches (2 × 1300 g, named as PC and PC + GC) in sterile conditions (laminar flow cabinet Polon KLVS-1, Poznań, Poland). The commercial starter cultures were added to the mixtures 1) PC: 0.5 g of MST Cheese-Tek^®^ and 0.5 g of PC^®^; 2) PC + GC: 0.5 g of MST Cheese-Tek^®^, 0.5 g of PC^®^ and 0.5 g of GEO^®^. The inoculated mixtures when then ground in a domestic mixer to obtain a homogenous consistency (approximately 5 min). After homogenization and inoculation, the mixtures were dispensed into sterile plastic Camembert cylindrical forms (Serowar, Szczecin, Poland) layered down with a gauze (200 ± 1 g of mixture per one form), formed finally six individual cheese analogues for each variant (6 PC and 6 PC + GC). The samples were allowed to ferment at room temperature (25 ± 1 °C) for 24 h. At the end of fermentation, the samples were removed from the forms and the gauze was slightly removed. Then each sample was evenly salted (1.5 g of NaCl per sample) and the samples were transferred to a climatic chamber (Binder FD15, Tuttlingen, Germany) and placed on cheese maturation mats. During 14 days (12 ± 1 °C, 90% RH (relative humidity)), the samples were turned daily. On day 14, the samples were wrapped in polyethylene-covered paper (0.06 mm thickness, Serowar, Szczecin, Poland) and the conditions have been changed (6 ± 1 °C, 90% RH), and the samples were allowed to mature for the next 14 days.

### 2.3. Microbiological Analyses

During the overall storage, samples (10 g) were collected and diluted with 90 mL of sterile physiological saline (0.9% NaCl), and serial dilutions were prepared [32]. Lactic acid bacteria counts were determined on MRS (de Man, Rogosa and Sharpe) medium (Merck, Darmstad, Germany) after incubation at 37 °C under anaerobic conditions for 72 h, whereas fungal counts were determined on Rose Bengal Agar at 25 °C for 72 h. The enumeration of microorganisms was performed in triplicate and the viable cell counts were expressed as CFU/g of the samples.

### 2.4. Extracts Preparation and Determination of Their Total Polyphenolic Content (TPC), Total Flavonoid Content (TFC) and Reducing Sugars Content (RCS)

Prior to extraction, the samples were lyophilized for 24 h (chamber pressure 0.190 mbar, shelf temperature T_min_ = −35 °C, T_max_ = 20 °C, condenser temperature −85 °C) in Beta 2–8 LSCplus lyophilizer (Martin Christ Gefriertrocknungsanlagen GmbH, Osterode am Harz, Germany). Lyophilized samples (1 g) were extracted in 40 mL of methanol in ultrasonic bath (Elmasonic S30H, Elma Schmidbauer GmbH, Singen, Germany) for 10 min, then centrifuged at 14,000 rpm/min for 10 min at 20 °C (Centrifuge 5418 Eppendorf, Warsaw, Poland) and filtered through a 0.22-µm nylon membrane filters (Sigma-Aldrich, Darmstadt, Germany). The obtained clear fluids were used for further analyses. The total polyphenolics and total flavonoids content of each supernatant were determined as described by Tong et al. [39]. To determine TPC, the supernatants (100 µL) were mixed with 6 mL of distilled water and 0.5 mL of Folin–Ciocalteu’s reagent. After 3 min, 1.5 mL of saturated Na_2_CO_3_ solution was added and the mixture was incubated for 30 min in darkness at 40 °C. The absorbance of the mixture was measured at 765 nm (UV-Vis Thermo Scientific Evolution 220 spectrophotometer). The concentration of TPC was calculated as mg of gallic acid equivalents (GAE) per g of sample (mg GAE/g). The determination of TFC consisted of mixing 250 µL of supernatants with 1 mL of distilled water and 75 µL of 5% NaNO_2_ solution. After 5 min, 75 µL of 10% AlCl_3_ solution was added, and the mixture was allowed to stand for 6 min before the addition of 250 µL of 1 M NaOH. The total volume mixture was made up to 3 mL with distilled water, and then the absorbance was measured at 510 nm (UV-Vis Thermo Scientific Evolution 220 spectrophotometer). Quercetin was used for a calibration curve, and the results were expressed as mg of quercetin equivalents (QE) per g of the sample (mg QE/g). The reducing sugars content (RSC) was determined by DNS (3,5-dinitrosalicylic acid) method [32]. A total of 10 g of DNS was dissolved in 200 mL of distilled water by continuous stirring, then slowly 16 g of NaOH (dissolved first in 150 mL of distilled H_2_O) was added. The mixture was incubated at 50 °C with stirring to obtain a clear solution. Then 403 g of potassium sodium tartrate tetrahydrate was added in small portions. The mixture was filtered using a paper filter and the volume was made up to 1000 mL with distilled water. One milliliter of supernatant was mixed with 1 mL of 0.05 m acetate buffer (pH 4.8), and 3 mL of DNS reagent was added, then vigorously shaken. The mixtures were incubated in boiled water for 5 min then cooled at room temperature. Then, the absorbance values were recorded at 540 nm (UV-Vis Thermo Scientific Evolution 220 spectrophotometer, Waltham, MA, USA). Glucose in acetate buffer was used for a calibration curve.

### 2.5. Determination of Reducing Power and Radical Scavenging Activity

To determine the reducing power, the methanolic supernatants (500 μL) were placed in a tube, to which 1.25 mL of phosphate buffer solution (0.2 M, pH 6.6), as well as 1.25 mL of 1% potassium ferricyanide solution were added. After incubation at 50 °C for 20 min, 1.25 mL of trichloroacetic acid solution were added to the tube. 1.25 mL of supernatant obtained by centrifugation at 3000 rpm for 10 min was diluted with 1.25 mL of deionized water. Finally, 0.25 mL of 0.1% ferric chloride solution was added to complete the assay. The absorbance was determined at 700 nm which represented the reducing power.

The DPPH and ABTS radical scavenging activity were determined according to the procedures as described in previous study [32]. In brief, the DPPH radical scavenging activity was determined by mixing one milliliter of the methanolic supernatants with 1 mL of 0.01 mM DPPH methanolic solution. The absorbance was measured at 517 nm. Three mL of ABTS^·+^ solution were mixed with 50 µL of the methanolic supernatants and the absorbance was measured at 734 nm.

Superoxide (O_2_^−^) scavenging activity was carried out based on pyrogallol oxidation inhibition following the methodology of Ye et al. with a slight modification [40]. Three mL of 50 mmol/L (pH 8.2) Tris-HCl buffer were mixed with 1 mL of the methanolic supernatants. These mixtures were mixed with a pyrogallol solution (0.3 mL, 7 mmol/L, preheated to 25 °C) and allowed to react for exactly 4 min, then 1 mL od 10 mmol/L of HCl was added to terminate the reaction, and absorbance was measured at 318 nm (Thermo Scientific Evolution 220 spectrophotometer). The O_2_^−^ scavenging rate was calculated from the formula:

(1) $${\%O}_{2}^{\;-\;}\;\mathrm{inhibition}=\left[1\;-\;\frac{\left(A_{1}\;-{\;A}_{1}^{\prime }\right)}{A_{0}}\right]\times 100$$

where A_1_ is the absorbance of the mixture in the presence of the sample, $A_{1}^{\prime }$ is the absorbance of water replacing the reaction agent; A_0_ is the absorbance of water replacing the sample solution.

The hydroxyl (^·^OH) scavenging assay was carried out based on method of Ye et al. [40]. One mL of methanolic supernatants and 1.5 mL of ortophenantroline solution (0.005 mmol/L) were mixed with 2 mL of phosphate buffer (pH 7.4, 0.05 mol/L). Then 1 mL of FeSO_4_ solution (0.0075 mol/L) was added and then mixed with 1 mL of H_2_O_2_ (0.1%), and finally supplemented with distilled water to a total volume of 10 mL. The reaction solution was kept at 37 °C for 1 h in darkness, then the absorbance was measured at 536 nm. The ortophenatroline solution without H_2_O_2_ addition (replaced by 1 mL of methanol) served as a blank. ^·^OH scavenging rate was be calculated using formula:

(2) $$\%{}^{\cdot }\mathrm{OH}\;\mathrm{inhibition}=\left[\frac{A_{2}\;-{\;A}_{1}}{A_{0}\;-{\;A}_{1}}\right]\times 100$$

where A_0_ is the ortophenatroline solution without H_2_O_2_ addition, A_1_ is the absorbance in presence of 1 mL of methanol instead of the sample, and A_2_ is the absorbance in the presence of the sample.

### 2.6. Determination of Total Solids Content (TSC), Protein Content (PC), Total Free Amino Acids Level (TFAAL), Ash Content (AC), pH, and Titrable Acidity (TA)

Total solids (no. 968.11), protein (no. 99120), and ash (no. 94546) contents were measured according to AOAC standard methods. A multiplication factor of 6.38 was used to convert nitrogen percentage to protein percentage [41]. To determine pH and TA, during the overall storage, samples (10 g) were collected and diluted with 90 mL of sterile physiological saline (0.9% NaCl), then measured directly at 25 °C using a pH-meter (CP-411, Elmetron, Zabrze, Poland). The TA determination consisted of mixing 10 mL of prepared dilutions with 10 mL od distilled water, and titrated with 0.01 M NaOH solution, using phenolophtalein (0.1%, w/v in 95% ethanol) as an indicator.

Total free amino acids level (TFAAL) was performed according to Barac et al. [42] with a slight modification. Lyophilized samples (1 g) were extracted in 40 mL of distilled water in ultrasonic bath (Elmasonic S30H, Elma Schmidbauer GmbH, Singen, Germany) for 10 min, then centrifuged at 14,000 rpm/min for 10 min at 20 °C (Centrifuge 5418 Eppendorf, Warsaw, Poland) and filtered through a 0.22 µm nylon membrane filters (Sigma-Aldrich, Darmstadt, Germany). A quantity of 1 mL of the filtrates were mixed with 2 mL of a Cd-ninhydrin reagent (0.8 g ninhydrin was dissolved in a mixture of 80 mL ethanol and 10 mL glacial acetic acid, followed by the addition of 1 g CdCl_2_ dissolved in 1 mL of distilled water). The mixtures were vortexed and heated at 84 °C for 5 min and cooled in ice-water, and the absorbance was determined at 507 nm. The results were expressed as milligram Gly per gram of lyophilized sample by reference to a standard curve which was first prepared using glycine at various concentrations (0.050–0.500 mg Gly/mL water).

### 2.7. Determination of Oil Content (OC) and Oxidative Stability

Chloroform-methanol (2:1 v/v) extraction was performed on the homogenized products based on method of Blight and Dyer [43]. Next, in the chloroform phase the peroxide value [44], anisidine value [45], acid value [46], iodine value [47] and total oxidation value (TOTOX) were determined. Oil content was determined by chloroform evaporation at 100 °C.

### 2.8. Texture Profile Analyses

Texture profiles were performed at room temperature using a Zwick/Roell 2,5 Z equipment (Zwick/Roell, Ulm, Germany), equipped with a cylindrical probe (diameter 40 mm). The samples were analyzed directly, penetration rate into the samples was 10 mm/s, and the penetration depth was 25 mm. From the results of the force-time curves, the firmness, gumminess, chewiness, cohesiveness and hardness were calculated.

### 2.9. Statistical Analysis

All determinations were carried out in triplicate, and all data were expressed as mean ± standard deviation (SD). Statistical significance was determined using an analysis of variance (two-way ANOVA) followed by NIR Fisher test. The values were considered as significantly different when *p* < 0.05. All analyses were performed with Statistica version 10 (StatSoft Polska, Kraków, Poland).

## 3. Results and Discussion

### 3.1. The Lactic Acid Bacteria and Fungi Survivability during the Ripening

**Figure 1:**
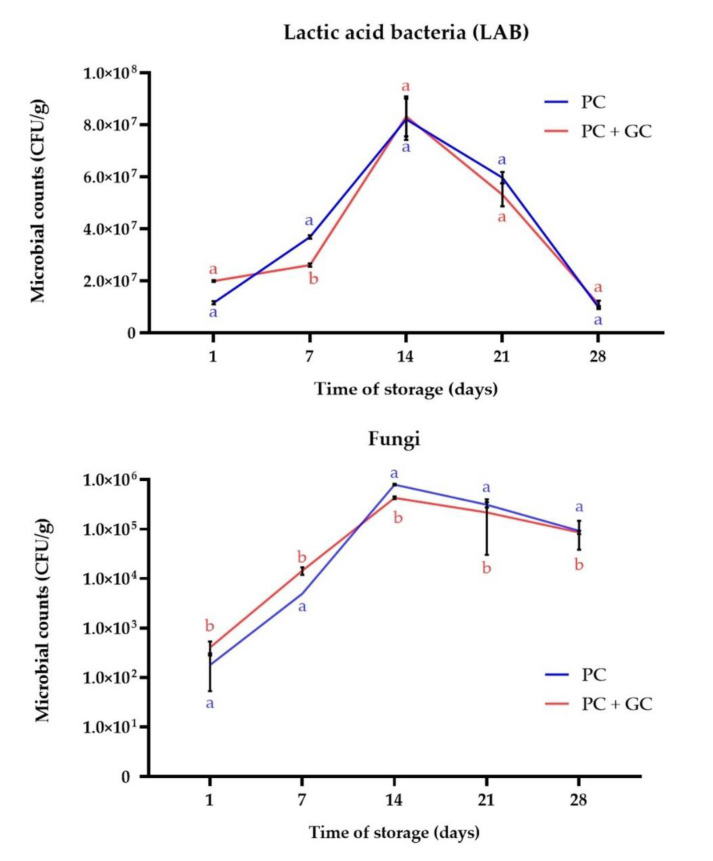
Lactic acid bacteria (LAB) and fungi counts during storage time of *Penicillium camemberti* (PC) and PC + *Geotrichum candidum* (GC) samples. Values are means ± standard deviation of triplicate determinations. Means with different lowercase are significantly different at *p* < 0.05.

**Figure 2:**
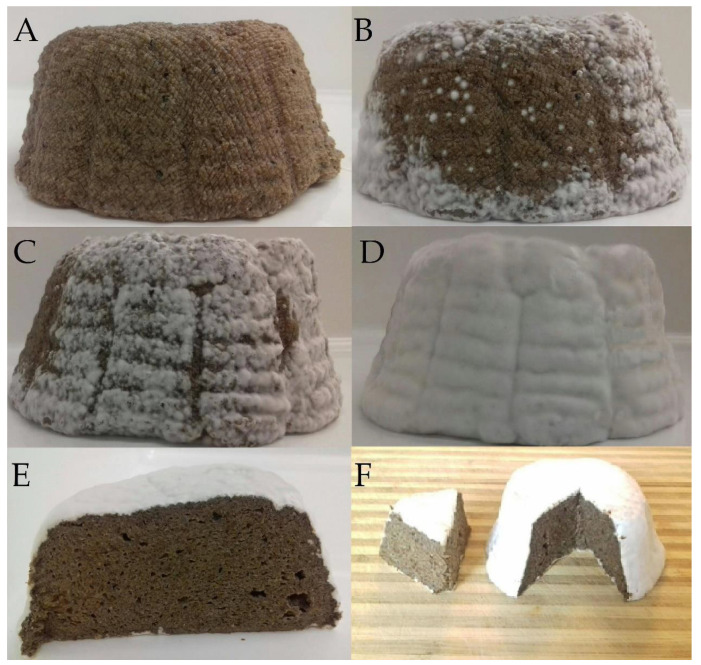
Representative photographs of sample PC + GC after 1 day (**A**), 7 days (**B**), 14 days (**C**), 21 days (**D**) and 28 days (**E**,**F**).

As presented in Figure 1, the formulations were an excellent matrix to develop non-dairy products since LAB and fungi survivability was high over ripening period. There were no visual differences between variants, and representative photographs of PC + GC sample during maturation are presented in Figure 2. After fermentation the bacterial counts in PC and PC + GC were 1.16 × 10^7^ ± 0.06 CFU/g and 2.00 × 10^7^ ± 0.03 CFU/g, respectively. Since day 21 a one log decrease of the LAB level was observed in sample PC (*p* < 0.05). It is reported that *G. candidum* may produce growth factors, that stimulate the function of bacteria in cheese, which through their enzymatic activity are also essential in cheese ripening [48]. The most difficult tasks in the functional cheese production is survival of bacteria during long ripening period. Therefore, 10^6^ CFU/g is the most widely accepted minimum concentration of LAB in food [9]. In general, at any time, the bacterial counts were maintained in the samples over level >10^6^ CFU/g, which may be attributed to prebiotic fiber content. In fact, HadiNezdah et al. demonstrated that flaxseed fiber acts as good prebiotic, enhancing the LAB growth in kefir model [49]. High survivability of LAB as well as yeast was observed in previous studies using FOC to produce kefir-like beverage [32] and yogurt-like plant milk [38]. Additionally, Chen et al. [14] reported high survivability of LAB in cashew cheese-analogues. *P. camemberti* is strictly aerobic, therefore it grows only on the cheese surface [50]. Indeed, a formation of a white crust was observed during PC and PC + GC maturation (Figure 2). It is generally agreed that this mold plays an important proteolytic and lipolytic role in the overall ripening process [50]. Currently, *G. candidum* is systematically added to ripening starters due to better knowledge about its growth and development, as well as its capacity to metabolize bitter peptides produced by *P. camemberti* [50]. The higher viable population of fungi in sample PC + GC recorded during the mixed culture compared to PC (*p* < 0.05) clearly highlight a synergistic interaction between both species. Indeed, the synergistic effect and mutualism (including increased secretion of hydrolytic enzymes) of *P. camemberti* and *G. candidum* when co-cultured was previously reported [26,28,51]. This indicates that the use of the two species together can lead to better substrate utilization and increased biotransformation productivity.

### 3.2. The Changes of Total Solids Content, Ash Content, pH, Titrable Acidity, Protein Content and Free Amino Acids Level

As expected, the physicochemical properties of FOC were modified by the microbial activity. Average values of total solids content (TSC), ash content (AC), pH, titrable acidity (TA), protein content (PC) and total free amino acids level (TFAAL) are summarized in Table 1. Classically, ripening of milk-based cheeses is an example of a process of complex microbial maturation, in which biochemical and microbiological changes take place in cheese mostly through the metabolism of residual lactose, lactate and citrate besides proteolysis and lipolysis, however a pivotal role is also played by the indigenous enzymes of milk and milk clotting enzyme (rennin) [9,19]. Generally, the results reveal that there was a gradual loss in the moisture content of PC and PC + GC during storage (*p* < 0.05), which is linked with water evaporation [52]. This result is in line with results of Schlesser et al. [53] and higher than reported by Diarra et al. [18] who reported 30% moisture content of ripened cheese-like product from peanut. A significant reduction of pH and increase of TA was observed after fermentation (*p* < 0.05). The reduction of pH is attributed to the production of organic acids (mainly lactic acid) by LAB, as the acidification of cheese-milk is their well-known function on cheese production [54]. In fact, a similar reduction of pH was reported from FOC fermented with kefir and yogurt cultures [32,38], as well as fermented cashew [2]. Since day 7 a significant increase of pH and decrease of TA was observed (*p* < 0.05). This observation is linked with fungal activity. Growth of *P. camemberti* and *G. candidum* is accompanied by medium alkalization, resulting from the assimilation of organic acids as carbon substrates and the release of ammonia due to amino acids deamination, the consequence of which is further increase of pH [24,26,28]. The proteinases of *P. camemberti* are activated by the increasing of pH and they migrate slowly into the cheese matrix [55]. Moreover, *G. candidum* consumes lactate for its growth, and it was supposed that this yeast consumed lactate diffusing from the core to the rind, causing the increase of pH and decrease of TA, which is in line with observations of Leclercq-Perlat [50]. Indeed, on day 28 a highest pH (*p* < 0.05) was observed in sample PC + GC (7.92 ± 0.01) in comparison with sample PC (7.35 ± 0.01). The protein content of the raw materials as well as in samples during ripening was also investigated. During the ripening period some fluctuations in PC were observed (*p* < 0.05). A decrease of PC was noticed in sample PC on 28 day, whereas in sample PC + GC a protein content decrease trend was observed since day 7, which may be attributed to proteolytic and peptidolytic activity.

It was found that TFAAL of both products significantly increased during the ripening, which indicates directly progressive proteolysis (*p* < 0.05). Upon fermentation, microbial proteases are released and degraded to a certain extent the proteins in a composite food matrix [10]. It was noticed, that proteolysis was highly related to the growth of *G. candidum* and *P. camemberti* known from their proteolytic and peptidolytic activities [50]. The release of amino acids is carried out by peptidases and proteinases of the starter culture, which means that, depending on the microbial strains used, different concentration of free amino acids may be obtained [9]. In milk-based Camembert, *G. candidum* improves flavor quality by bitterness reduction as it hydrolyses low molecular weight peptides that originate from the casein by *P. camemberti* [26]. Indeed, significantly higher FAAL during ripening was observed in sample PC + GC during the ripening period (*p* < 0.05). Aziza et al. reported that free amino acid concentration increased gradually during ripening of cheese with mixed culture of *P. camemberti* and *G. candidum* [26]. Diarra et al. also reported high degree of proteolysis in peanut cheese-like product during ripening [18]. The production of free ammonia can be understood as the final step of proteolysis, and is linked with observed alkalization of PC and PC + GC [50]. Furthermore, increased level of amino acids may enhance organoleptic properties such as aroma, taste and flavor, since amino acids are precursors of these features [9].

### 3.3. The Changes of Total Phenolic, Total Flavonoid and Reducing Sugars Contents

Table 2 presents the results of TPC, TFC and RSC determinations. A significant increase in RSC after fermentation in comparison to the non-fermented samples was observed (*p* < 0.05). The increased RSC may be linked with enzymatic hydrolysis of polysaccharides such as flaxseed fiber by microorganisms (particularly LAB), to obtain energy required for growth, thus generating higher amounts of polysaccharides derivatives [32]. Similar observations have been reported in previous studies using FOC to produce kefir-like beverage [32] and yogurt-like plant milk [38]. However, on 7 days a decrease of RSC in both samples was noticed, which may be attributed to polysaccharides derivatives consumption by microorganisms, required for maintain metabolic activity and was reported in previous findings [32,38]. Plant matrices are rich in non-starch polysaccharides, i.e., arabinoxylans, β-glucans, cellulose, lignin, etc., and often exist in composite structures with other small molecular weight compounds, e.g., phenolics, flavonoids, and minerals. During the fermentation process, the pH is decreased due to production of organic acids (mainly lactic acid) and this may result in activation of various enzymes, either endogenous of the plants or bacterial. The enzymatic activity is responsible for biopolymers degradation, leading to cell wall degradation (softening). LAB make the food easily digestible, decreasing the level of high-chain carbohydrates and some indigestible poly- and oligo-saccharides [56]. This is of great importance from the sensorial, physicochemical and dietary point of view, because carbohydrates digestibility is related to many human health issues [10]. As a result of fermentation, a significant increase of TPC and TFC was noticed (*p* < 0.05). In this study, the highest TPC was detected in sample PC + GC on day 28 (35.78 ± 0.02 mg GAE/g). The highest TFC was observed in sample PC + GC on day 14 (12.52 ± 0.05 mg QE/g). However, some decrease of TFC was noticed in sample PC + GC since day 21. Flaxseed is one of excellent sources for phenolic substances. Main types of phenolic substances in flaxseed include phenolic acid compounds, lignans and flavonoid compounds. Phenolic acid compounds, for example ferulic acid, gallic acid, chlorogenic acid, hydroxycinnamic acid glucosides, *p*-coumaric acid glucosides, etc., were detected in defatted flaxseed [12,33,36]. The effect of fermentation on the TPC and antioxidant activity of plant matrices has been highlighted in numerous studies [10]. Indeed, in previous studies the increase of TPC and TFC of FOC as a result of fermentation (attributed to delinking of some phenolic compounds that were bounded to proteins and cell wall carbohydrates) have been reported [32,38]. It is of particular importance, because phenolic compounds need to be in a soluble form to enter the human blood circulation system and bring about their antioxidant properties [10].

### 3.4. The Changes of Antioxidant and Radical Scavenging Activities

The antioxidant capacity and radical scavenging activities of FOC-based products are summarized in Table 3. As can be seen, the RP increased significantly (*p* < 0.05) by fermentation. However, some decrease of DPPH, O_2_^−^ and ^·^OH radical scavenging activity was observed at the end of ripening period, conversely an increase of ABTS and radical scavenging activity was noticed during storage time (*p* < 0.05). Those differences may be attributed to different radicals acting mechanisms. For instance, ABTS method measures both electron- and proton-transfer reactions, sensing lipophilic and hydrophilic compounds [57]. Generally, the antioxidant activity of the products is linked with microbial activity, production and liberation of certain bioactive compounds which demonstrate reducing power and react with free radicals to stabilize and terminate radical chain reactions [32]. Barac et al. [42] reported that reducing power of white-brined cheeses increased as a result of proteolysis and liberation of bioactive compounds. Moreover, some LAB strains are able to reduce the oxidative processes of dairy fermented products [58]. Strong radical scavenging activity of low molecular weight peptides from different cheeses is well known. Additionally, it is known that almost all amino acids can interact with highly energetic free radicals [42]. Similarly, the formation of bioactive peptides in plant matrices with antioxidant activity as a result of fermentation was previously reported [32]. Thus, based on TPC, TFC and DP results it is reasonably to conclude, that the total antioxidant activity of PC and PC + GC was manifold. The high antioxidant activity of FOC-based products and their ability to scavenge free radicals have been reported [32,38]. Based on those results is also tempting to suggest, that the antioxidant capacity of the FOC-based products was related to oxidative stability of flaxseed oil, which is in line with findings of other authors [59].

### 3.5. The Changes of Oil Content and Oxidative Stability

Based on the analysis of the quality of extracted oil from the tested products summarized in Table 4, it was noticed that during storage and ripening of FOC-based products, there was no intensive increase in the overall oxidation level (they were oxidatively stable) However, in PC + GC sample, TOTOX values were approximately 2-times higher over the storage period (*p* < 0.05). In the case of primary oxidation products (PV), after 3 weeks there was a significant increase in both products (*p* < 0.05). Secondary oxidation products (AV) were at a similar level throughout the experiment. After preparation, the average fat content in the samples was 3.0 ± 0.3% and some oil content decrease was noticed (*p* < 0.05). On day 28 lower oil content was observed in PC sample (2.4 ± 0.2%), in comparison to PC + GC sample (2.7 ± 0.3%). However, the oil content in both products is much lower than in classic Camembert cheese, therefore, FOC-based products can be considered as a low-fat alternative. *P. camemberti* and *G. candidum* are able to produce lipases which are determining enzymes for the aroma/flavor of mold-ripened cheeses [19,28]. Although some fluctuations were observed, the oil in the obtained products was characterized by a high content of unsaturated acids during ripening, which was confirmed by the initial and final determination of iodine (IV) number. There was no decrease in the degree of fat unsaturation during storage. This stability may be linked with liberation of some antioxidant compounds (such as polyphenolics and flavonoids known to stabilize the flaxseed oil) due to microbial activity [59]. However, in the case of determination of free fatty acids by means of the acid number in the extracted oil, a significant decrease was observed during storage (*p* < 0.05), which may be attributed to lypolitic activity and viability of microorganisms in the tested samples [9,25,28].

### 3.6. The Textural Changes

Cheese texture is the result of a complex interaction factors such as cheese composition, manufacturing process and undergoes continuous changes induced by biochemical activities that occur throughout the ripening process [15]. In Camembert cheese deacidification results on formation on creamy and homogenous appearance during ripening [24]. As can be seen in Table 5, the textural changes of PC and PC + GC samples during ripening were noticed. A noticeably differences between samples were observed since 7 day. For instance, a significantly higher hardness was noticed in sample PC during storage period (*p* < 0.05). However, no significant differences were observed regarding cohesiveness (*p* > 0.05). The complex changes observed during the ripening period can be attributed to water loss, plant cell wall degradation (indicated by formation of RSC) and other factors such as pH changes [8,15,60]. Additionally, protein content plays an important role in texture formation due to proteins gelation properties [54]. In this context, the changes in the texture of the cheese during ripening can be explained by the constant breakdown and re-establishment of the protein bonds [16]. Ong et al. reported that cheese with higher protein level showed higher hardness [54]. Indeed, a lower hardness and chewiness was observed in PC + GC sample in comparison to PC sample (*p* < 0.05), which may be attributed to lower protein level and higher TFAAL.

## 4. Conclusions

Taking into account the increasing complexity of the needs of different groups of consumers, including vegan/vegetarian, and subjects with medical problems (such as intolerance/allergy to dairy products), an approach in this work was applied to obtain a novel bioactive Camembert cheese analogue from flaxseed oil cake substrate, using LAB and individual (*P. camemberti*) or mixed (*P. camemberti* + *G. candidum*) fungal starter cultures, and describe the changes that occur during ripening. Our study provides an extensive body of evidence that obtained products are good sources of polyphenols, with undisputed antioxidant effects. It was demonstrated that the bioactivity as well as the physicochemical properties depend on the starter culture used. Given the growing global interest in healthy food the richness in biologically active compounds makes the flaxseed oil cake Camembert-like products a promising functional food from a nutritional point of view. There is a potential for these products to be used where non-dairy alternatives are desired. However, further studies are necessary to improve the industrial process, and in particular to sensorial characteristics.

## Figures and Tables

**Table 1:** pH, titrable acidity (TA), total solids content (TSC), ash content (AC), protein content (PC), and total free amino acids level (TFAAL) of the samples.

Time of Storage (Days)						
Sample	0	1	7	14	21	28
pH						
PC	6.50 ± 0.01 ^Aa^	6.18 ± 0.02 ^Ba^	6.31 ± 0.01 ^Ca^	6.73 ± 0.02 ^Da^	6.78 ± 0.00 ^Ea^	7.35 ± 0.01 ^Fa^
PC + GC	6.45 ± 0.01 ^Ab^	6.01 ± 0.01 ^Bb^	6.25 ± 0.05 ^Cb^	6.82 ± 0.01 ^Db^	7.04 ± 0.01 ^Eb^	7.92 ± 0.01 ^Fb^
TA (g lactic acid/100 g)						
PC	0.12 ± 0.02 ^Aa^	0.72 ± 0.01 ^Ba^	0.68 ± 0.00 ^Ca^	0.67 ± 0.04 ^Ba^	0.52 ± 0.07 ^Da^	0.38 ± 0.00 ^Ea^
PC + GC	0.10 ± 0.01 ^Aa^	0.80 ± 0.01 ^Bb^	0.72 ± 0.01 ^Ca^	0.60 ± 0.01 ^Db^	0.42 ± 0.05 ^Eb^	0.22 ± 0.08 ^Fb^
TSC (%)						
PC	25.64 ± 0.40 ^Ab^	25.89 ± 2.06 ^Aa^	27.83 ± 0.33 ^Ba^	27.86 ± 1.29 ^Ba^	26.72 ± 0.37 ^ABa^	23.72 ± 0.39 ^Ca^
PC + GC	24.40 ±1.03 ^ABCc^	26.86 ± 1.54 ^Ba^	27.86 ± 1.15 ^Ba^	25.15 ± 0.35 ^ABb^	25.25 ± 0.56 ^ABb^	23.36 ± 0.48 ^Ca^
AC (%)						
PC	1.63 ± 0.01 ^Ab^	1.88 ± 0.02 ^Ba^	2.05 ± 0.03 ^Ba^	2.34 ± 0.03 ^Ca^	2.00 ± 0.06 ^Ba^	2.10 ± 0.06 ^BCa^
PC + GC	1.54 ± 0.04 ^Ab^	2.30 ± 0.03 ^Bb^	1.99 ± 0.04 ^Ca^	2.23 ± 0.35 ^BCb^	2.19 ± 0.01 ^BCa^	2.05 ± 0.07 ^Ca^
PC (g/100 g)						
PC	4.50 ± 0.01 ^Ab^	5.11 ± 0.01 ^Ba^	4.65 ± 0.03 ^Ca^	5.84 ± 0.01 ^Da^	6.10 ± 0.04 ^Ea^	4.60 ± 0.00 ^Fa^
PC + GC	4.66 ± 0.00 ^Ac^	4.35 ± 0.00 ^Bb^	5.17 ± 0.03 ^Cb^	5.12 ± 0.00 ^Db^	4.73 ± 0.01 ^Eb^	4.48 ± 0.00 ^Fb^
TFAAL (mg Gly/g)						
PC	6.84 ± 0.00 ^Aa^	7.44 ± 0.00 ^Ba^	8.25 ± 0.00 ^Ca^	8.28 ± 0.00 ^Da^	8.40 ± 0.05 ^Ea^	9.36 ± 0.01 ^Fa^
PC + GC	7.02 ± 0.00 ^Ab^	7.49 ± 0.00 ^Bb^	8.39 ± 0.01 ^Cb^	8.42 ± 0.01 ^Db^	8.57 ± 0.02 ^Eb^	9.40 ± 0.01 ^Fb^

Values are means ± standard deviation of triplicate determinations. Means with different lowercase in the same column are significantly different at *p* < 0.05. Means with different uppercase in the same raw are significantly different at *p* < 0.05.

**Table 2:** Total polyphenolics content (TPC), total flavonoids content (TFC) and reducing sugars content (RSC) changes of the samples during storage.

Time of Storage (Days)						
Sample	0	1	7	14	21	28
TPC (mg GAE/g)						
PC	14.68 ± 0.08 ^Aa^	16.84 ± 0.18 ^Ba^	17.60 ± 0.20 ^Ca^	24.46 ± 0.00 ^Da^	27.00 ± 0.66 ^Ea^	31.02± 0.03 ^Fa^
PC + GC	14.44 ± 0.11 ^Aa^	16.40 ± 0.11 ^Bb^	18.49 ± 0.43 ^Cb^	30.34 ± 0.11 ^Db^	32.87 ± 0.11 ^Eb^	35.78 ± 0.02 ^Fb^
TFC (mg QE/g)						
PC	7.67 ± 0.22 ^Aa^	7.90 ± 0.05 ^Aa^	9.83 ± 0.05 ^Ba^	12.52 ± 0.05 ^Ca^	11.00 ± 0.27 ^Da^	9.24 ± 0.00 ^Ea^
PC + GC	7.55 ± 0.05 ^Ab^	7.93 ± 0.03 ^Ba^	9.79 ± 0.10 ^Ca^	9.24 ± 0.00 ^Cb^	10.03 ± 0.05 ^Db^	9.72 ± 0.10 ^Cb^
RSC (mg/g)						
PC	22.12 ± 0.03 ^Aa^	31.92 ± 0.04 ^Ba^	26.40 ± 0.05 ^Ca^	20.27 ± 0.02 ^Da^	19.92 ± 0.03 ^Ea^	18.99 ± 0.03 ^Ea^
PC + GC	23.19 ± 0.00 ^Aa^	33.22 ± 0.02 ^Bb^	21.10 ± 0.01 ^Cb^	19.58 ± 0.01 ^Db^	19.54 ± 0.01 ^Eb^	16.44 ± 0.01 ^Eb^

Values are means ± standard deviation of triplicate determinations. Means with different lowercase in the same column are significantly different at *p* < 0.05. Means with different uppercase in the same raw are significantly different at *p* < 0.05.

**Table 3:** DPPH, ABTS, O_2_^−^, ^·^OH radical scavenging activity and reducing power (RP) of the samples during storage.

Time of Storage (Days)						
Sample	0	1	7	14	21	28
DPPH inhibition (%)						
PC	77.46 ± 0.00 ^Aa^	94.00 ± 0.20 ^Ba^	94.11 ± 0.16 ^Ba^	93.07 ± 0.33 ^Ca^	89.64 ± 0.16 ^Da^	81.55 ± 0.21 ^Ea^
PC + GC	76.23 ± 0.00 ^Ab^	93.76 ± 0.11 ^Ba^	94.23 ± 0.04 ^Ca^	93.30 ± 0.47 ^Da^	87.38 ± 0.19 ^Eb^	80.36 ± 0.07 ^Fb^
ABTS inhibition (%)						
PC	42.84 ± 0.00 ^Aa^	52.97 ± 0.00 ^Ba^	55.74 ± 0.00 ^Ca^	57.43 ± 0.00 ^Da^	66.93 ± 0.00 ^Ea^	57.63 ± 0.09 ^Fa^
PC + GC	45.18 ± 0.09 ^Ab^	56.10 ± 0.00 ^Bb^	56.43 ± 0.09 ^Cb^	56.47 ± 0.09 ^Cb^	61.65 ± 0.09 ^Db^	59.04 ± 0.00 ^Eb^
O_2_^−^ inhibition (%)						
PC	40.59 ± 0.90 ^Aa^	54.93 ± 0.74 ^Ba^	59.13± 0.86 ^Ca^	67.90 ± 0.99 ^Da^	65.31 ± 0.12 ^Ea^	54.17 ± 0.16 ^Ba^
PC + GC	41.65 ± 0.41 ^Aa^	59.13 ± 0.99 ^Bb^	52.66 ± 0.35 ^Cb^	65.52 ± 0.06 ^Db^	64.65 ± 1.20 ^Eb^	54.87 ± 0.40 ^Fa^
RP 700 nm						
PC	0.139 ± 0.02 ^Aa^	0.176 ± 0.01 ^Ba^	0.233 ± 0.01 ^Ca^	0.208 ± 0.03 ^Da^	0.276 ± 0.01 ^Ea^	0.168 ± 0.01 ^Fa^
PC + GC	0.140 ± 0.01 ^Aa^	0.161 ± 0.02 ^Bb^	0.183 ± 0.02 ^Cb^	0.182 ± 0.01 ^Cb^	0.202 ± 0.01 ^Db^	0.171 ± 0.01 ^Eb^
^·^OH inhibition (%)						
PC	35.48 ± 0.02 ^Aa^	40.17 ± 0.03 ^Ba^	53.60 ± 0.00 ^Ca^	63.17 ± 0.00 ^Da^	77.77 ± 0.02 ^Ea^	67.49 ± 0.05 ^Fa^
PC + GC	34.57 ± 0.01 ^Ab^	48.57 ± 0.01 ^Bb^	53.45 ± 0.01 ^Cb^	61.45 ± 0.05 ^Db^	71.38 ± 0.01 ^Eb^	69.48 ± 0.01 ^Fb^

Values are means ± standard deviation of triplicate determinations. Means with different lowercase in the same column are significantly different at *p* < 0.05. Means with different uppercase in the same raw are significantly different at *p* < 0.05.

**Table 4:** Oil content and oil oxidative stability of the samples during storage.

Time of Storage (Days)						
Sample	0	1	7	14	21	28
Oil Content (%)						
PC	3.00 ± 0.03 ^Aa^	3.30 ± 0.04 ^Ba^	3.61 ± 0.05 ^Ca^	3.11 ± 0.02 ^Da^	2.91 ± 0.01 ^Ea^	2.41 ± 0.04 ^Fa^
PC + GC	3.00 ± 0.03 ^Aa^	2.90 ± 0.05 ^Bb^	3.03 ± 0.02 ^Ab^	2.91 ± 0.05 ^Bb^	2.81 ± 0.05 ^Cb^	2.70 ± 0.05 D^b^
Peroxide Value (mg O/100 g)						
PC	4.59 ± 0.74 ^Aa^	11.32 ± 3.30 ^Ba^	17.70 ± 0.33 ^Ca^	8.19 ± 1.43 ^Aa^	12.38 ± 0.31 ^Ba^	55.18 ± 2.32 ^Da^
PC + GC	6.89 ± 0.99 ^Aa^	16.42 ± 1.01 ^Bb^	22.06 ± 2.12 ^BCb^	21.56 ± 0.48 ^BCb^	23.34 ± 2.35 ^Cb^	40.08 ± 0.00 ^Db^
Anisidine Value (-)						
PC	2.17 ± 0.33 ^Aa^	2.24 ± 0.23 ^Aa^	3.71 ± 0.59 ^Ba^	3.62 ± 0.58 ^Ba^	9.00 ± 0.05	3.95 ± 0.35 ^Ba^
PC + GC	1.02 ± 0.21 ^Ab^	8.79 ± 0.82 ^Bb^	7.73 ± 0.15 ^Cb^	10.18 ± 0.05 ^Db^	6.57 ± 0.62 ^Eb^	2.95 ± 0.29 ^Fb^
TOTOX (-)						
PC	2.28 ± 0.31 ^Aa^	2.53 ± 0.26 ^Aa^	4.17 ± 0.59 ^Ba^	3.84 ± 0.54 ^Ba^	9.32 ± 0.06 ^Ca^	3.33 ± 0.30 ^Da^
PC + GC	1.20 ± 0.23 ^Ab^	9.22 ± 0.74 ^Bb^	8.30 ± 0.21 ^Cb^	10.74 ± 0.07 ^Db^	7.18 ± 0.68 ^Eb^	10.13 ± 0.44 ^Fb^
Acid Value (mg NaOH/g)						
PC	65.96 ± 0.07 ^Aa^	88.12 ± 0.44 ^Ba^	84.68 ± 0.14 ^Ca^	79.08 ± 0.64 ^Da^	62.43 ± 0.52 ^Ea^	59.31 ± 0.43 ^Fa^
PC + GC	75.66 ± 0.83 ^Ab^	74.99 ± 0.44 ^Bb^	82.14 ± 0.67 ^Cb^	80.37 ± 0.18 ^Db^	78.59 ± 0.36 ^Eb^	58.72 ± 0.38 ^Fa^
Iodine Value (g/100 g)						
PC	156.95 ± 1.03 ^Aa^	173.37 ± 2.67 ^Ba^	171.84 ± 3.36 ^Ba^	189.99 ± 2.81 ^Ca^	204.08 ± 3.28 ^Da^	183.59 ± 0.00 ^Ea^
PC + GC	160.60 ± 7.43 ^Aa^	160.21 ± 1.12 ^Ab^	182.80 ± 0.27 ^Bb^	194.48 ± 3.33 ^Cb^	200.49 ± 1.15 ^Cb^	173.47 ± 2.11 ^Db^

Values are means ± standard deviation of triplicate determinations. Means with different lowercase in the same column are significantly different at *p* < 0.05. Means with different uppercase in the same raw are significantly different at *p* < 0.05.

**Table 5:** Textural changes of the samples during storage.

Time of Storage (Days)						
Sample	0	1	7	14	21	28
Springness (N)						
PC	1.33 ± 0.15 ^Aa^	1.45 ± 0.08 ^Aa^	1.64 ± 0.21 ^Ba^	1.55 ± 0.22 ^Ca^	2.01 ± 0.11 ^Da^	2.08 ± 0.18 ^Da^
PC + GC	1.34 ± 0.10 ^Aa^	1.42 ± 0.10 ^Aa^	1.58 ± 0.92 ^Bb^	1.83 ± 0.18 ^Cb^	1.87 ± 0.16 ^Cb^	1.66 ± 0.16 ^Da^
Gumminess (N)						
PC	0.75 ± 0.05 ^Aa^	0.87 ± 0.12 ^Aa^	1.38 ± 0.09 ^Ba^	1.53 ± 0.17 ^Ca^	1.72 ± 0.12 ^Da^	1.92 ± 0.32 ^Ea^
PC + GC	0.72 ± 0.03 ^Aa^	0.78 ± 0.21 ^Aa^	0.82 ± 0.09 ^Ab^	1.02 ± 0.10 ^Bb^	1.45 ± 0.32 ^Cb^	1.23 ± 0.10 ^Bb^
Chewiness (N)						
PC	1.01 ± 0.20 ^Aa^	1.50 ± 0.14 ^Aa^	1.65 ± 0.27 ^Aa^	2.34 ± 0.29 ^Ba^	3.45 ± 0.68 ^Ca^	3.17 ± 0.64 ^Ca^
PC + GC	0.99 ± 0.23 ^Aa^	1.18 ± 0.55 ^Aa^	1.20 ± 0.09 ^Aa^	1.86 ± 0.23 ^Ba^	2.70 ± 0.13 ^Cb^	2.05 ± 0.22 ^Bb^
Cohesiveness (N)						
PC	0.54 ± 0.07 ^Aa^	0.48 ± 0.05 ^Ba^	0.35 ± 0.02 ^Ca^	0.27± 0.02 ^Da^	0.30 ± 0.04 ^CDa^	0.25 ± 0.04 ^Da^
PC + GC	0.56 ± 0.02 ^Aa^	0.46 ± 0.01 ^Ba^	0.34 ± 0.07 ^Ca^	0.27± 0.05 ^Da^	0.27 ± 0.01 ^Da^	0.25 ± 0.04 ^Da^
Hardness (N)						
PC	2.11 ± 0.44 ^Aa^	2.20 ± 0.16 ^Aa^	2.47 ± 0.55 ^Aa^	3.86 ± 0.20 ^Ba^	3.92 ± 0.74 ^Ba^	5.37 ± 0.26 ^Ca^
PC + GC	2.10 ± 0.52 ^Aa^	2.15 ± 0.23 ^Aa^	2.28 ± 0.49 ^Aa^	2.85 ± 0.15 ^Bb^	3.66 ± 0.79 ^Ca^	3.62 ± 0.57 ^Cb^

Values are means ± standard deviation of triplicate determinations. Means with different lowercase in the same column are significantly different at *p* < 0.05. Means with different uppercase in the same raw are significantly different at *p* < 0.05.
